# Ferroptosis regulator SLC7A11 is a prognostic marker and correlated with PD-L1 and immune cell infiltration in liver hepatocellular carcinoma

**DOI:** 10.3389/fmolb.2022.1012505

**Published:** 2022-10-04

**Authors:** Yimin Liang, Shijie Su, Zhaoxia Lun, Zishao Zhong, Weifeng Yu, Guihua He, Qi Wang, Jing Wang, Suiping Huang

**Affiliations:** ^1^ The Second Clinical College of Guangzhou University of Chinese Medicine, Guangzhou, China; ^2^ Science and Technology Innovation Center, Guangzhou University of Chinese Medicine, Guangzhou, China; ^3^ Institute of Clinical Pharmacology, Guangzhou University of Chinese Medicine, Guangzhou, China; ^4^ Department of Gastroenterology, The Second Affiliated Hospital of Guangzhou University of Chinese Medicine, Guangzhou, China

**Keywords:** ferroptosis, SLC7A11, PD-L1, prognosis, immune cell infiltration, liver hepatocellular carcinoma

## Abstract

**Background:** Liver hepatocellular carcinoma (LIHC) is a complicated disease with poor survival and lack of viable treatment options. The roles of ferroptosis and immunotherapy in LIHC are increasingly prominent, but the interplay of ferroptosis with the tumor microenvironment (TME) in LIHC is currently under-investigated.

**Methods:** In this study, we analyzed normal liver tissues and tumor tissues from the TCGA and GTEx databases to obtain differentially expressed ferroptosis-related genes (FRGs). We then clustered LIHC based on the expression levels of selected FRGs and acquired distinct subtypes with significant heterogeneity regarding survival prognoses, PD-L1 expression, and immune cell infiltration. The correlation of those FRGs with TME in LIHC and pan-cancer analysis was also investigated. GO functional annotations and KEGG pathway analyses were performed to investigate the potential reactions of the obtained differentially expressed genes (DEGs). Further external validation was performed using microarrays on the GEO database and the key ferroptosis regulator SLC7A11 expression between LIHC and normal cells was detected by Western blotting.

**Results:** A large proportion of genes were upregulated in the LIHC group. Among three clusters, cluster 3 had the worst prognosis combined with the highest PD-L1 expression and was positively correlated with various immune cells. Subsequently, survival analysis and Cox regression analysis screened out SLC7A11 as an independent prognostic factor in LIHC featured strong PD-L1 expression and unfavorable survival time. We filter out SLC7A11 as an independent prognostic signature in LIHC patients with strongly associated PD-L1 expression and unfavorable survival probability. In the pan-cancer analysis, high expression of SLC7A11 showed poor overall survival in seven cancers, while the correlation between immune checkpoints (ICs) and SLC7A11 varied by cancer type, indicating the potential therapeutic effects of SLC7A11 in cancers other than LIHC. Western blot was further employed to verify the expression of SLC7A11 in LIHC *in vitro*.

**Conclusion:** Ferroptosis and TME synergistically play key roles in oncogenesis and progression of LIHC, and SLC7A11 can be used as a predictive biomarker for customized immunotherapy.

## Introduction

Liver hepatocellular carcinoma (LIHC) is the most prevalent kind of primary malignancy of the liver, accounting for approximately 75% (Xu et al., 2018), and the fourth leading cause of cancer-related mortality globally, generally on the background of chronic hepatitis (Mak et al., 2020). Most LIHC patients are diagnosed at the middle-late stage, making them ineligible for drastic surgery. However, tyrosine kinase inhibitor sorafenib, the sole FDA-approved first-line molecular targeted medication for the treatment of advanced LIHC, has demonstrated promising outcomes in clinical practices (Gordan et al., 2020). Accumulating body of evidence (Dixon et al., 2014; Nie et al., 2018; Xia et al., 2019) has reported that sorafenib can trigger a novel and effective regulatory cell death—ferroptosis while using the iron chelator DFX can protect LIHC cells from the cytotoxicity of sorafenib (Louandre et al., 2013). Ferroptosis, characterized by the accumulation of iron-dependent lipid peroxides and reactive oxygen species to lethal levels, has been implicated in various cancer types, most notably LIHC (Galluzzi et al., 2018; Nie et al., 2018). Liu (Liu et al., 2020) also found that the ferroptosis potential index positively correlated with tumor stage and prognostic outcomes. These results suggested the therapeutic potential of ferroptosis in LIHC.

Although sorafenib-induced ferroptosis extended survival time in LIHC patients, frustratingly severe adverse events and rising drug resistance discounted the effectiveness to some extent (Nie et al., 2018). Therefore, innovative combination treatment is desperately needed to enhance clinical outcomes. The liver is an organ with innate immune tolerance, and TME affects the prognosis and progression at large (Xu et al., 2018). Huang et al. found that macrophage-associated cytokines might be a non-invasive biomarker for predicting PD-L1 levels in LIHC patients (Huang et al., 2021). PD-L1 is a vital IC of tumor immunity highly expressed in LIHC tumor tissues and peripheral immune cell components such as kupffer cells and CD8^+^ T cells (Wu et al., 2009). High PD-L1 expression increases the likelihood of tumor recurrence, metastasis, and even mortality from cancer (Xu et al., 2018). In recent years, immune checkpoint blocking therapy has made breakthroughs in LIHC immunotherapy, especially a phase III trial of atezolizumab and bevacizumab targeting anti-PD1/PD-L1 antibodies and reprogramming TME exhibited better results than sorafenib (Galle et al., 2021). However, given these implications, whether ferroptosis plays a synergistic role with PD-L1 and the immune microenvironment in LIHC is still largely elusive.

In this work, we explored the differentially expressed FRGs in tumor and normal tissues of LIHC and obtained three subtypes through consistent clustering analysis with diverse prognoses, which not only correlated with PD-L1 expression but also varied in immune cell infiltration. Furthermore, SLC7A11, the highly expressed ferroptosis regulator in cluster 3, was linked to a worse outcome and had a substantial connection with PD-L1 (CD274) and both survival and Cox regression analysis indicated that it played a prognostic role in LIHC. In addition, SLC7A11 was also shown to be strongly expressed in multiple tumor types in pan-cancer analysis, interacting with multiple ICs, including PD-L1. This study aims to provide insights into ferroptosis-based immunotherapy and better management for LIHC patients.

## Materials and methods

### Data availability

RNA-seq data containing 50 normal liver tissues and 371 LIHC tissues and related clinicopathologic information were downloaded from The Cancer Genome Atlas (TCGA) database’s Genomic Data Commons (GDC) portal (https://portal.gdc.cancer.gov/) in December 2021. FRGs were derived from the systematic analysis of the aberrances and functional implications of ferroptosis in cancer by Liu (Liu et al., 2020). Meanwhile, 226 normal samples from the GTEx database (https://gtexportal.org/home/datasets), and GSE60502 combined with GSE62232 (28 normal tissues and 99 LIHC tissues) were downloaded from the Gene Expression Omnibus database (GEO; https://www.ncbi.nlm.nih.gov/geo/) were available for further validation about the expression level of the SLC7A11 gene. In the pan-cancer analysis of SLC7A11, RNA-seq data for 33 cancer types and their corresponding clinical details were also derived from TCGA.

### Bioinformatics analysis

The differentially expressed genes (DEGs) of LIHC were identified using the “limma” package of R software, and the significant expression levels of DEGs were visualized with “pheatmap” and “ggplot2” packages. Gene ontology (GO) functional entries, namely biological process (BP), cellular component (CC), and molecular function (MF), along with Kyoto Encyclopedia of Genes and Genomes (KEGG) pathway enrichment analyses, were performed on the upregulated and downregulated DEGs, respectively, to explore related biological functions based on the “ClusterProfiler” package. Principal component analysis (PCA) was presented by “ggord” package. The “Consensus Cluster Plus” package was then adopted for consistency assessment (the maximum cluster was 6, and 80% of the samples were conducted 1,000 times). Overall survival (OS) and disease-free survival (DFS) prognostic analyses on different clusters were carried out by “survival” and “survminer” packages. We conducted survival analysis on SLC7A11 in Kaplan-Meier (KM) plotter online database (http://kmplot.com/). To further detect the status of the TME, CIBERSORT was utilized to measure the proportion of immune cell infiltration across distinct clustering subtypes. Univariate and multivariate Cox regression analyses were conducted on multiple clinical features to determine the independent prognostic significance of SLC7A11 in LIHC. *p*-values, hazard ratio (HR), and 95% confidence interval (CI) for the results were visualized in the form of a forest plot based on “forestplot” package. Furthermore, pan-cancer analysis was applied to explore the differential expressions of SLC7A11 among various cancers and its potential in PD-L1 immunotherapy.

### Verification of the expression of SLC7A11 *in vitro*

The LIHC cell lines HepG2, Hep3B, BEL-7402, BEL-7407, and MHCC97L, and the normal liver cell line L02 were generously provided by Tang’s Lab. Normal and hepatocellular carcinoma cell lines were cultured in 10%FBS/DMEM at 37 °C with 5% CO_2_. The cells in the logarithmic growth phase were collected for Western blotting. The cell supernatant was obtained by low-temperature sonication and centrifugation (4°C, 12,000*xg*, 15 min). The protein concentration in the cell supernatant was determined by ultraviolet spectroscopy. The total protein amount of each lane was 40 μg in the 10% SDS-PAGE gel. PVDF membranes were blocked with 5%w/v skim milk. The primary antibody was incubated overnight at 4°C; the secondary antibody was incubated for 1 h at room temperature. Image acquisition of the target bands using enhanced chemiluminescence immunoblotting (ECL, Bio-Rad, Japan). Anti-SLC7A11 (Abmart, T57046, 1:1,000), anti-*β*-actin (Affinity, AF7018, 1:1,000); Goat Anti-Rabbit IgG (H + L) HRP, S0001, 1:1,000); Goat Anti-Mouse IgG (H + L) HRP (Affinity, S0002, 1:1,000).

### Statistical analysis

Statistical testing and model building were mainly performed by R software (version 4.0.2) and GraphPad Prism 8.0. Student’s t-test was used to analyze the expressions of SLC7A11 in 25 pairs of LIHC and adjacent tissues groups; one-way ANOVA was used to compare whether the differences in the two groups differed by the expression level of SLC7A11. The Wilcox and the Kruskal–Wallis tests were used to compare two or more groups. The log-rank test of the Kaplan-Meier method was carried out for survival analysis. The Cox proportional hazards model was conducted for multivariate regression analysis to screen out independent prognostic characteristics. Predictive efficiency of SLC7A11 for 1-, 3-, and 5-years OS were evaluated using the receiver operating characteristic (ROC) curves in “timeROC” package. The association between ICs and ferroptosis was evaluated using Spearman’s rank correlation coefficient. *p* < 0.05 was accepted as indicative of statistically significant differences.

## Results

### Differential expression of FRGs between LIHC and adjacent normal tissues

**FIGURE 1:**
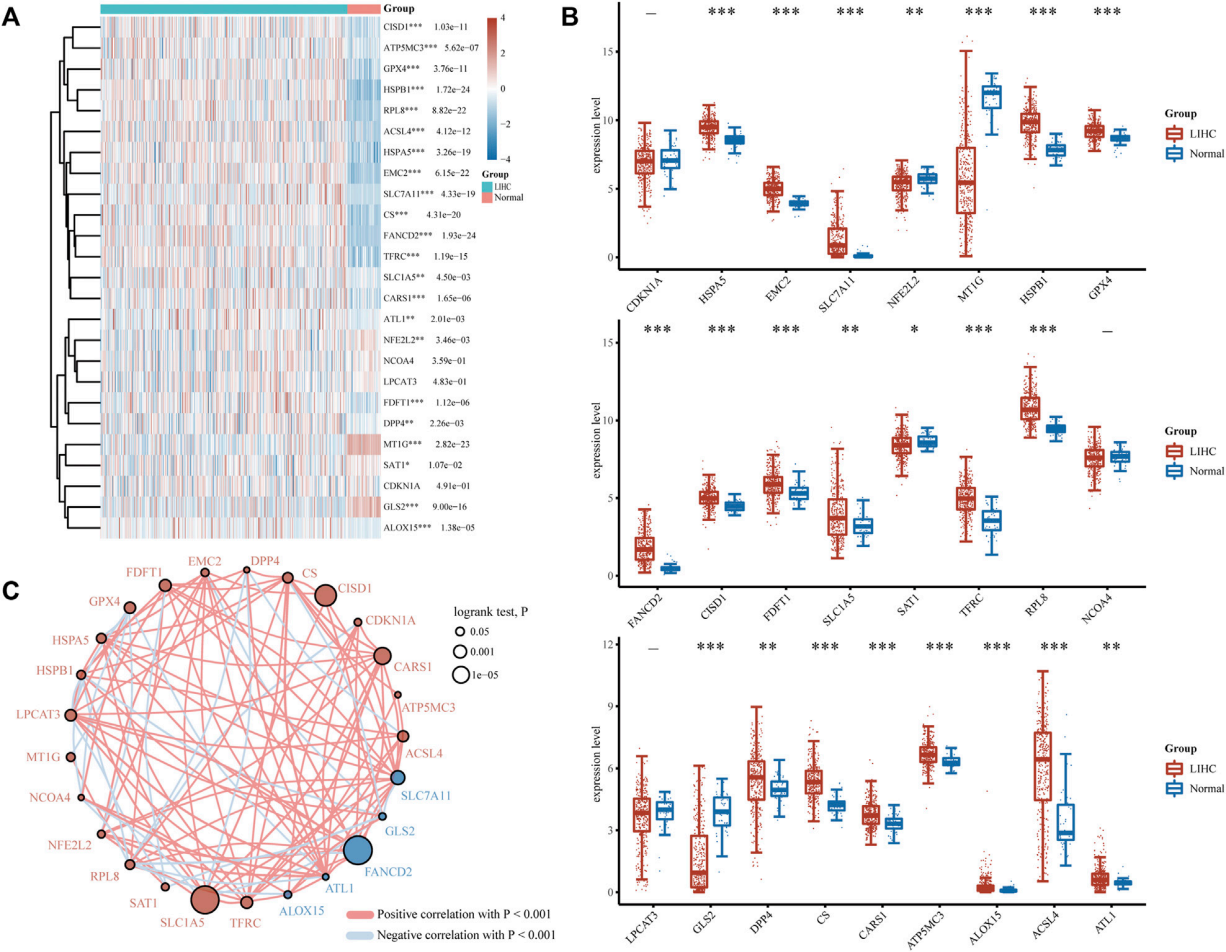
Divergent expression distribution and correlation of FRGs in LIHC and normal tissues. **(A)** FRG expression heatmap and **(B)** boxplot between the LIHC and normal group. **(C)** Spearman correlation and prognostic network of FRGs in LIHC. The red and blue dots, respectively, signify a poor and good prognosis. The larger their circles are, the smaller the log rank test. *P* of prognosis is, and vice versa. *p < 0.05, **p < 0.01, and ***p < 0.001.

FRGs were designated as a set of 25 genes identified as playing essential roles in regulating ferroptosis in a prior study (Dixon et al., 2012). To determine the predictive molecular mechanism of ferroptosis regulators in LIHC, we performed a comprehensive analysis of the gene expression profiles of 371 LIHC patients and 50 normal individuals via the TCGA database, and found that the expression levels of screened FRGs in tumor and normal controls varied (Figures 1A,B). As is illustrated, HSPA5, EMC2, SLC7A11, HSPB1, GPX4, FANCD2, CISD1, FDFT1, SLC1A5, TFRC, RPL8, DPP4, CS, CARS1, ATP5MC3, ALOX15, ACSL4 and ATL1 were the upregulated ferroptosis regulators (*p* < 0.05), and the NFE2L2, MT1G, SAT1 and GLS2(*p* < 0.05) were the downregulated ones. However, no significant differences were found between the LIHC and normal tissues regarding the expression of CDKN1A, NCOA4, and LPCAT3 (*p* > 0.05). Furthermore, the correlation and prognosis analysis network revealed that the vast majority of ferroptosis regulators were typically positively correlated with LIHC (Figure 1C). These findings highlight the underlying roles of ferroptosis in LIHC development and progression. As a result, further comprehensive study on the biological function and dysregulation of FRGs in LIHC will be helpful.

### FRGs expression levels of LIHC subtypes and different prognoses

**FIGURE 2:**
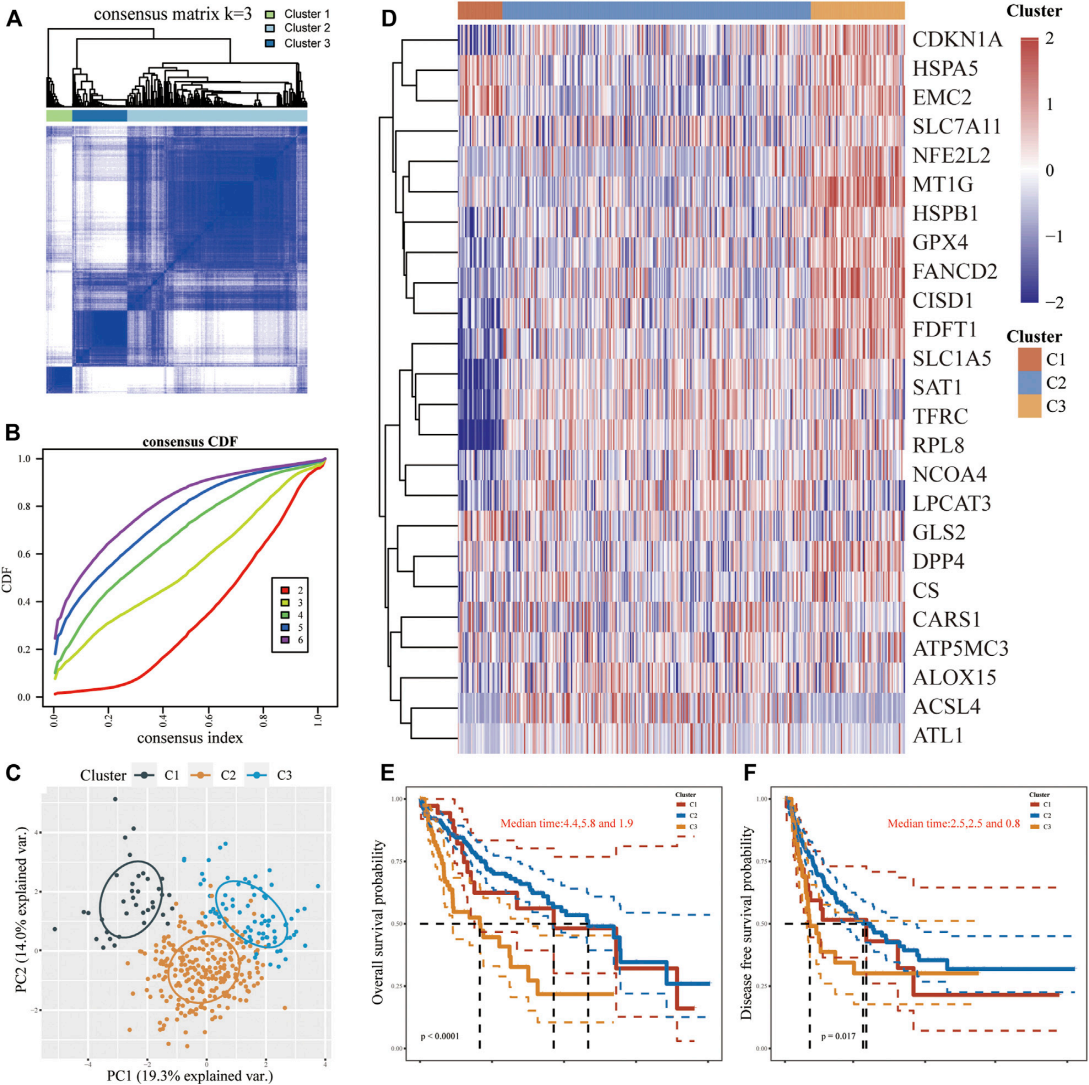
FRGs expression levels in different LIHC subtypes and their prognostic patterns. **(A,B)** Consistent clustering analysis on screening the LIHC subtypes. **(C)** PCA analysis was performed to clarify the difference in clusters. **(D)** Heatmap of FRGs expression levels in different LIHC subtypes. **(E,F)** The OS and DFS among three subtypes.

According to the expression microarray of FRGs in tumor and normal samples, using an unsupervised consistent clustering analysis method, we took consensus CDF and delta area into account, and determined the optimal cluster as *k* = 3 (Figures 2A,B; Supplementary Figures S1A-I). Consequently, three diverse subtypes of 371 LIHC patients were classified, namely cluster 1 (*n* = 37), cluster 2 (*n* = 256) and cluster 3 (*n* = 78). The PCA analysis also perfectly confirmed this distinction (Figure 2C). In comparison to cluster 1/2, most FRGs (9/25) were notably expressed in cluster 3, while the expression levels of CDKN1A and NCOA4 in cluster 2 were significantly higher than in others. FRGs were weakly expressed in cluster 1 but exclusive to HSPB1, GPX4, and RPL8, which were higher than those among clusters. Whereas two FRGs (MT1G and SAT1) showed no significance in any clusters (Figure 2D). Furthermore, patients in cluster 3 were more prone to dismal OS (*p* < 0.0001) and DFS (*p* < 0.05) in contrast with the other two clusters (Figures 2E,F). Clinical baseline data of 371 patients showed that there were statistical differences between three subtypes in terms of survival status, tumor size and invasiveness, total clinical stage, and differentiation grade (*p* < 0.01), but no significant differences in lymph nodes dissemination and distant metastasis (*p* > 0.1, Supplementary Table S1). Intriguingly, more patients in cluster 2 appeared to harbor worse tumor staging, but statistics on mortality between clusters found cluster 3 to be the top, followed by cluster 1 and then cluster 2, supposing the cause may root in the maximum sample size in cluster 2. These outcomes suggested that clinical heterogeneity exists in subgroups of LIHC.

### The relevancy of FRGs between PD-L1 and TME in LIHC

**FIGURE 3:**
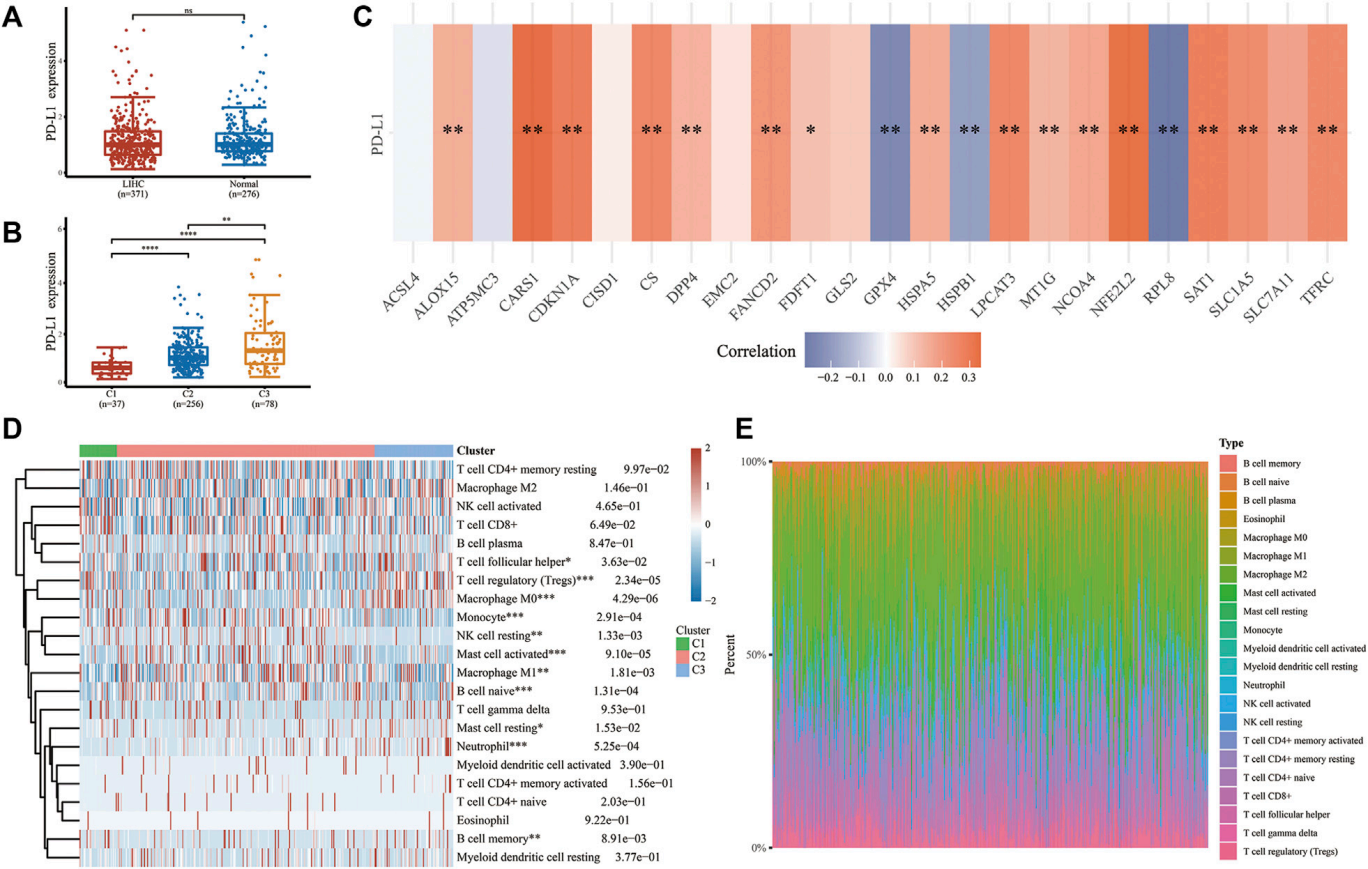
The relationship between PD-L1 and FRGs and the immune infiltration landscape of three subtypes in LIHC. **(A,B)** The expression differences of PD-L1 in LIHC and normal tissues and among different subtypes. **(C)** Correlation between PD-L1 and selected FRGs in TCGA cohort of LIHC. **(D)** Heatmap and proportional plotting graph **(E)** of immune cell abundance within three subtypes. *p < 0.05, **p < 0.01, ***p < 0.001, and ***p < 0.0001.

We investigated the character of FRGs in PD-L1 expression and TME to determine whether ferroptosis has immune efficacy in LIHC. Although the expressions of PD-L1 between tumor and normal tissues showed no statistically significant difference (Figure 3A), there were substantial differences within LIHC subgroups, with cluster 3 being the most overexpressed one, followed by cluster 2 and cluster 1 (Figure 3B). Coincidentally, different responsiveness to PD-L1 was also exploited in FRGs of LIHC (Figure 3C). Most regulators were positively correlated with PD-L1 expression (ALOX15, CARS1, CDKN1A, NFE2L2, SLC7A11, CS, DPP4, FANCD2, FDFT1, HSPA5, LPCAT3, MT1G, NCOA4, SAT1, SLC1A5, TEFC), while three regulators (GPX4, HSPB1, RPL8) were the opposite. Meanwhile, several FRGs (ACSL4, ATP5MC3, CISD1, EMC2, GLS2) did not respond to PD-L1. Furthermore, LIHC subtypes have immune microenvironment characteristics as assessed by the CIBERSORT algorithm. Cluster 3 possessed higher levels of memory B cell, T cell follicular helper, T cell regulatory (Tregs), macrophage M0, mast cell resting, and neutrophil infiltration. In contrast, cluster 1 and cluster 2 were more correlated with the B cell naive, NK cell resting, monocyte, mast cell activated, and macrophage M1 (Figures 3D,E; Supplementary Figure S2). Herein, we disclosed the potential of FRGs in the PD-L1 treatment of LIHC from the perspective of the immune microenvironment.

### SLC7A11 is the key FRG upregulated in LIHC

**FIGURE 4:**
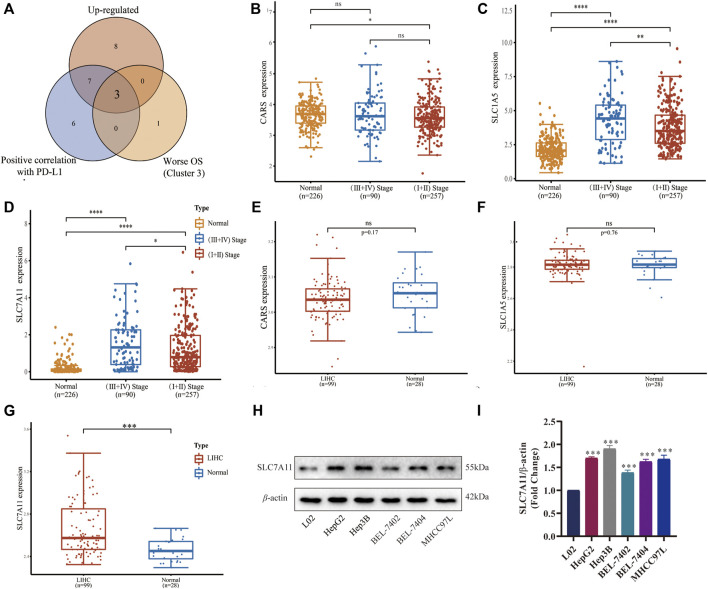
Expression and verification of a key ferroptosis regulator SLC7A11 in LIHC. **(A)** Venn diagram identified three upregulated FRGs associated with PD-L1 and worse prognoses. **(B–D)** Differential expression of CARS, SLC1A5, and SLC7A11 in different stages of tumor group and normal group in GSE60502. **(E–G)** Differential expression of CARS, SLC1A5, and SLC7A11 in the tumor group and normal group in GSE62232. **(H)** The expressions of SLC7A11 in different cell lines were detected by Western blot (*n* = 3). **(I)** Histogram of differential SLC7A11 expression on five LIHC cell lines. *p < 0.05, **p < 0.01, ***p < 0.001 and ****p < 0.0001.

Since the important roles of FRGs in different subtypes of LIHC have been illustrated above, we attempted to identify the major up-regulated FRGs with grim prognoses characterized with PD-L1 responsiveness. Then we found that CARS, SLC1A5, and SLC7A11 were three dominant ferroptosis regulators positively correlated with PD-L1 expression in LIHC and unfavorable OS (Figure 4A). We further compared the differences between tumor stages and normal tissues of the above-mentioned three FRGs in GSE60502 (Figures 4B–D), and the differences between tumor and normal tissues in GSE62232 (Figures 4E–G). Only SLC7A11 was highly expressed in the late-stage group compared with the normal and early-stage ones (*p* < 0.0001 and *p* < 0.05, respectively, Figure 4D), but also had a significant difference between LIHC and adjacent normal tissues (*p* < 0.001, Figure 4G). Later, we detected the expressions of SLC7A11 in different cell lines using Western blot (Figure 4H), and found SLC7A11 was highly expressed in LIHC cell lines HepG2, Hep3B, BEL-7402, BEL-7407, and MHCC97 L (*p* < 0.001, Figure 4I) compared with the normal control, indicating that SLC7A11 was the primary FRG implicated in the onset and development of LIHC.

### High exposure to SLC7A11 in LIHC is an effective independent prognostic factor

**FIGURE 5:**
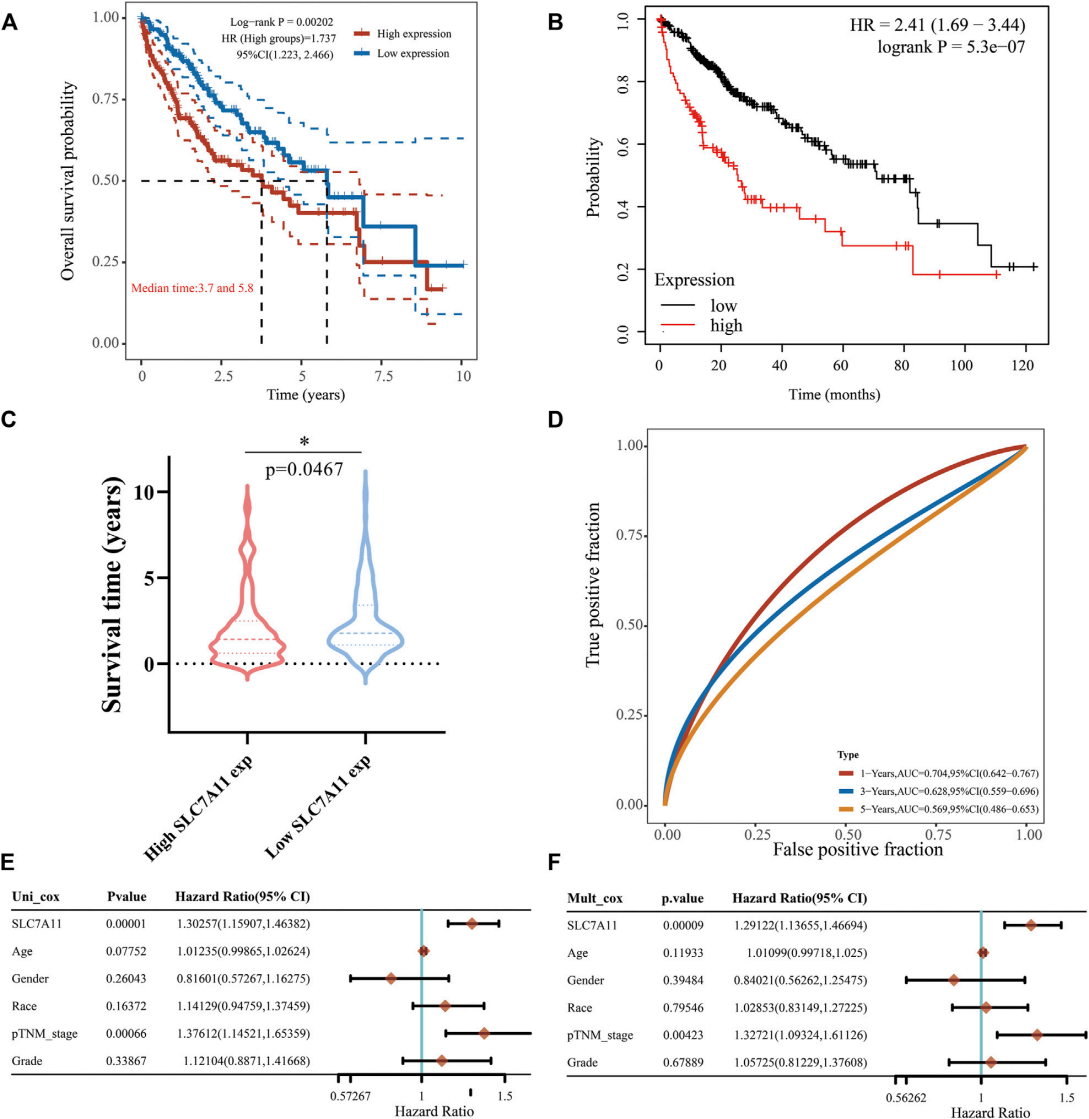
Effect of SLC7A11 expression difference on LIHC prognosis. **(A)** KM analysis for high and low SLC7A11 groups based on TCGA source data and the KM Plotter website **(B,C)** Differences in survival time between high- and low-SLC7A11 expression. **(D)** 1-, 3-, and 5-years survival prediction reliability of SLC7A11 as a prognostic factor. **(E,F)** Forest plots of univariate and multivariate Cox analyses for SLC7A11 and other clinical traits. *p < 0.05.

Later we focused on the impact of SLC7A11 expression on the prognoses of LIHC patients. The high-expression group of SLC7A11 had a poorer OS than the low-expression group (Figure 5A, HR = 1.737, *p* < 0.005), and this was also confirmed in the KM curve (Figure 5B, HR = 2.41, *p* < 0.0001). Similarly, the high SLC7A11 group in LIHC showed a shorter survival time compared with the low one (Figure 5C, *p* < 0.05). The ROC curves displayed the AUC values of SLC7A11 expression for predicting survival rate, which decreased sequentially at 1-, 3- and 5-years, but were all greater than 0.5, indicating that SLC7A11 was equipped with the prognostic potential (Figure 5D). Univariate Cox proportional hazard model revealed that SLC7A11 (HR = 1.30257, *p* = 0.00001), race (HR = 1.141,229, *p* = 0.16372), age (HR = 0.07752, *p* = 0.07752), tumor stage (HR = 1.37612, *p* = 0.00066) and grade (HR = 1.12104, *p* = 0.33867) were all shown to be risk factors for LIHC (Figure 5E). In addition, multivariate forest plot analysis further identified the expression of SLC7A11 (HR = 1.29122, *p* = 0.00009) as an independent risk factor affecting LIHC patients’ prognoses (Figure 5F). These all converged to the point that the high expression of SLC7A11 was a significant independent prognostic signature in LIHC.

### The landscape of SLC7A11 expression differences with related biological pathways and correlation with TME

**FIGURE 6:**
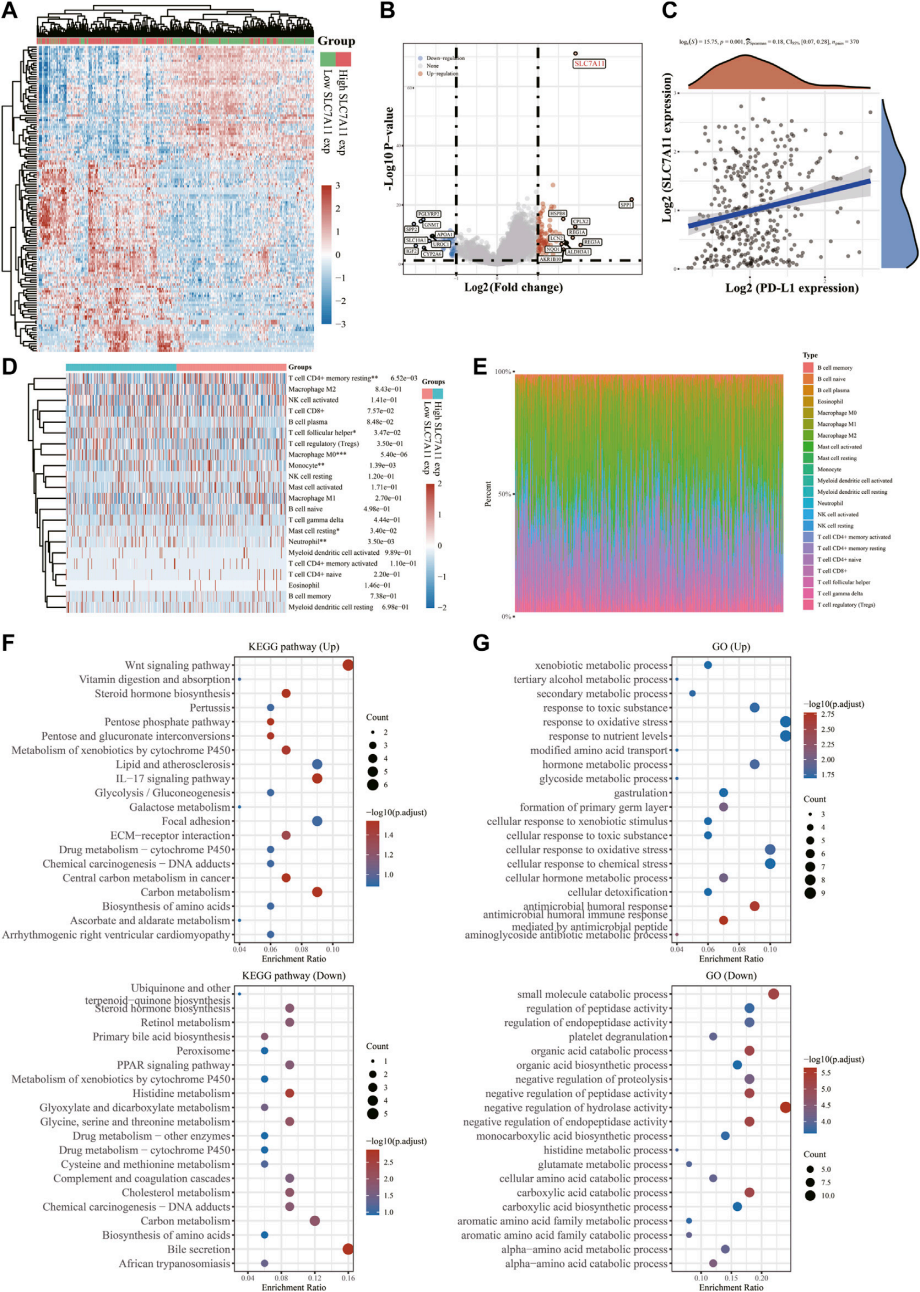
Differential expressions of SLC7A11 in LIHC and its correlation with PD-L1 and immune infiltration. **(A)** Heatmap of differences in high- and low-SLC7A11 expression groups. **(B)** Volcano plot of up- and down-regulated DEGs between the high- and low-SLC7A11 expression groups. **(C)** Correlation analysis of SLC7A11 and PD-L1 expression. **(D)** Immune cell infiltration profiles and relative proportions **(E)** in groups of different SLC7A11 expressions. **(F)** KEGG bubble charts of up- and down-regulated DEGs. **(G)** GO bubble charts of up- and down-regulated DEGs. *p < 0.05, **p < 0.01, and ***p < 0.001.

To further examine the underlying functions of SLC7A11 in LIHC, 371 samples were divided into two groups based on SLC7A11 expression levels (Figure 6A). We compared the DEGs in the two groups and discovered that SLC7A11, SPP1, REG3A, CPLX2, HSPB8, REG1A, LCN2, NQO1, AKR1B10 and ALDH3A1 were up-regulated genes, whereas SPP2, SLC10A1, IGF2, PGLYRP2, GNMT, APOA1, UROC1 and CYP2A6 were down-regulated ones (Figure 6B). Spearman correlation analysis exhibited a significant positive correlation between SLC7A11 and PD-L1 expression (*p* = 0.001, Spearman = 0.18), indicating that SLC7A11 may be a predictive biomarker for tumor immune checkpoint inhibitors (ICIs) therapy (Figure 6C). The CIBERSORT algorithm was then used to quantify immune infiltrating variations (Figure 6D, Supplementary Figure S3) and the proportion of 22 immune cells in low- and high-SLC7A11 expressing LIHC patients (Figure 6E, Supplementary Figure S3). T cell follicular helper, macrophage M0, mast cell resting, neutrophil enriched in SLC7A11 high expression group and T cell CD4^+^ memory resting, monocyte gained prominence in SLC7A11 low expression group (*p* < 0.05). GO entry and KEGG pathway enrichment analyses were used to identify relevant biological functions and pathways of DEGs in two samples with different expression levels of SLC7A11. KEGG results exhibited that up-regulated DEGs, including SLC7A11, were associated with the Wnt signaling pathway, central carbon metabolism in cancer, IL-17 signaling pathway, and other concerned pathways; however, down-regulated DEGs were engaged in bile secretion, histidine metabolism, cholesterol metabolism, retinol metabolism and other pathways related to liver physiological metabolism (Figure 6F). GO enrichment analysis suggested that up-regulated FRGs mainly focus on xenobiotic metabolic process, secondary metabolic process response toxic substance, tertiary alcohol metabolic process, etc. While the down-regulated ones prefer small molecular catabolic process, organic acid biosynthetic process, negative regulation of hydrolase activity and other biological processes (Figure 6G). These findings indicated the abnormalities and heterogeneity of immunological infiltration in SLC7A11 expression, which might serve as indicators and targets for immunotherapy, perhaps with important therapeutic ramifications.

### Prognostic value of SLC7A11 across 33 cancers and association with diverse ICs

**FIGURE 7:**
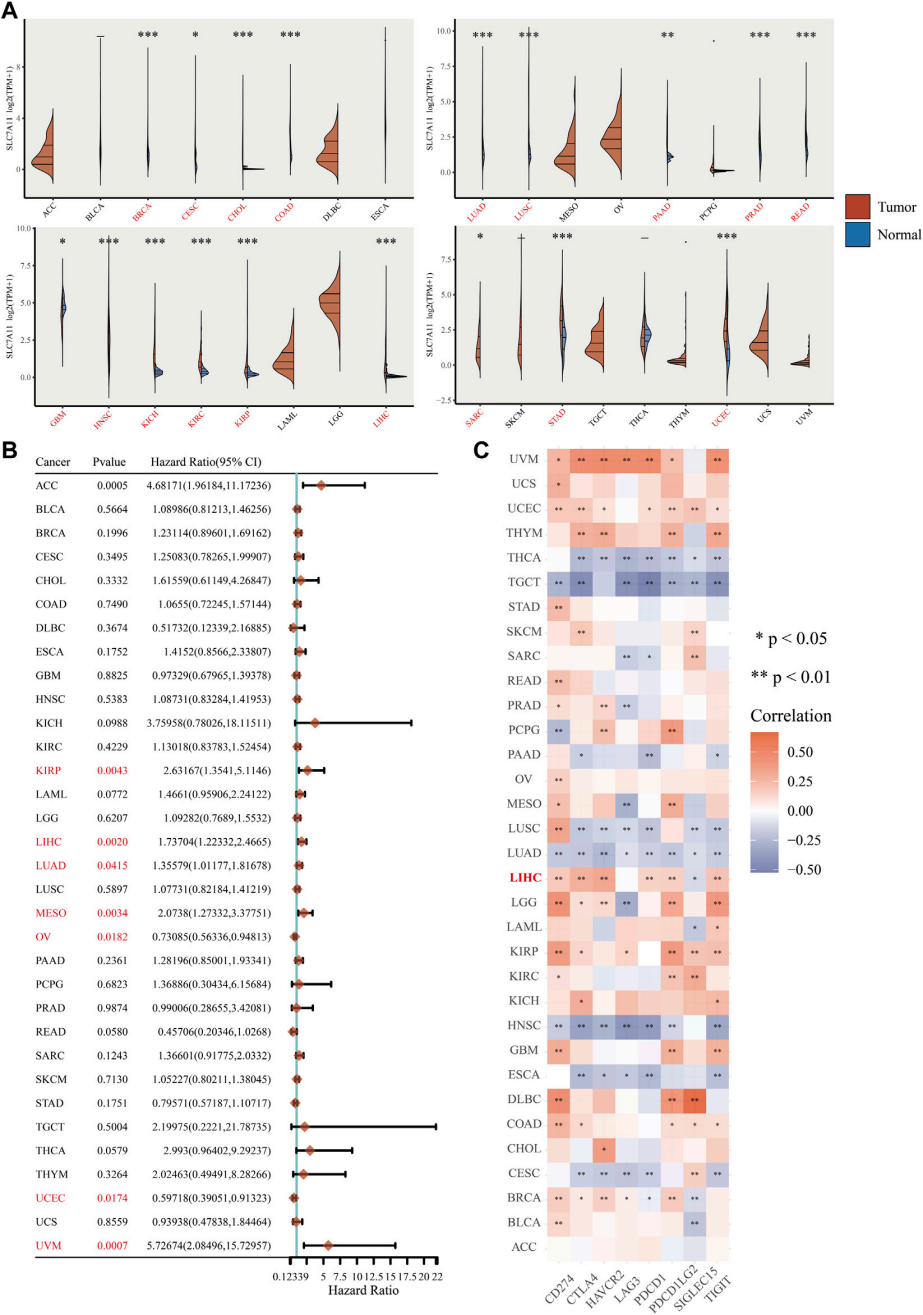
Association of SLC7A11 expression with prognoses and PD-L1 responses in multiple cancers. **(A)** Expression of SLC7A11 in normal and tumor tissues in 33 cancers. **(B)** The effect of high SLC7A11 expression on prognoses of various cancers. **(C)** Association of SLC7A11 with ICs in 33 cancer types. *p < 0.05, **p < 0.01, and ***p < 0.001.

Given the potential significance of the key ferroptosis regulator SLC7A11 in LIHC progression and treatment, we performed a pan-cancer analysis of SLC7A11 based on TCGA and GTEx databases to examine the similarities and differences in prognoses and IC responsiveness across 33 tumor types. Differential analysis of paired 26,772 tumor and normal tissue samples showed that SLC7A11 was upregulated in 17 cancers (BRCA, CESC, CHOL, READ, LUAD, LUSC, PRAD, HNSC, KICH, KIRC, KIRP, LIHC, PAAD, SARC, STAD, COAD and UCEC) but only downregulated in GBM (*p* < 0.05, Figure 7A). Cox regression analysis of 33 cancers showed that SLC7A11 expression was significantly associated with worse OS in seven cancers, including KIRP, LIHC, LUAD, MESO, OV, UCEC, and UVM (*p* < 0.05, Figure 7B). Besides, our research manifested the correlation of SLC7A11 with 8 IC genes, namely CD274 (also known as PD-L1), HAVCR2, LAG3, SIGLEC15, PDCD1LG2, CTLA4, PDCD1, and TIGIT in 33 cancer types. And SLC7A11 responded well to ICs in most cancers, particularly the robust performance with PD-L1 in LIHC (*p* < 0.01, Figure 7C). In conclusion, the potential correlation between SLC7A11 expression patterns and IC gene responses may help understand the therapeutic mechanisms of SLC7A11 in LIHC and other cancer types.

## Discussion

Ferroptosis, a novel iron-dependent programmed cell death bearing different fingerprints from another kind of programmed cell death, was initially described by Dixon in 2012 (Dixon et al., 2012). It has since been proven to offer intriguing promise in cancer therapies (Hassannia et al., 2019). Ongoing evidence has established that iron overload could exert toxic damage to the liver (Pantopoulos, 2018), and LIHC is vulnerable to ferroptosis, which has become a hotspot of clinical treatment and prognosis research (Capelletti et al., 2020). However, the underlying mechanism of ferroptosis regulators in the TME of LIHC is yet unknown. Therefore, we divided LIHC into three subtypes based on ferroptosis-related DEGs, and explored each subtype’s survival and prognosis traits as well as its interaction with the immune microenvironment. High expression of the key ferroptosis regulator SLC7A11 was found to be a major independent prognostic predictor of LIHC with characteristic heterogeneity of immune cell infiltration and PD-L1 expression. And on top of that, we unveiled the correlation of SLC7A11 predicting poor prognoses in various cancer types and association with multiple IC genes in the pan-cancer analysis.

The burgeoning development of molecular biology techniques and systematic biology provides technical support for the transformation from conventional histological classification to a more precise molecular profile, which is the foundation of individualized precise therapy (Malone et al., 2020). Given the complex heterogeneity of LIHC, patients may differ in disease progression, clinical efficacy, chemotherapeutic sensitivity and prognoses. Therefore, subtyping is imperative for the better identification of molecular-based strategies. Here, we performed cluster analysis on LIHC expression profiles and teased out three subtypes, which coincide with previous findings (Semaan et al., 2017), with cluster 3 having the highest PD-L1 expression and clearly poorer clinical stage and lethality than cluster 1/2. Many cancers have the ability of immune evasion, mainly by overexpressing PD-L1 on the tumor surface to dampen T cell attacks (Xu et al., 2018). Therefore, damaged and inactivated T cells might be detected in the LIHC patients, contributing to this cancer’s aggressiveness (Jia et al., 2015). This also meant the higher PD-L1 expression could be a possible biomarker to predict the sensitivity to anti-PD1/PD-L1 immunotherapy of patients in cluster 3.

TME-resident cytokines serve as the “battlefield” of immunotherapy and greatly impact tumor occurrence and metastasis, particularly when checkpoint-blocking therapies are used (Chen et al., 2019). Tfh and Tregs are both subsets of CD4^+^ helper T cells, and the expression of PD-L1 in LIHC causes Tfh depletion, which results in the inability to assist B cells to proliferate and produce antibodies in humoral immunity (Zhou et al., 2016). It has been well-established that up-regulated ICs increased the recruitment of numerous immunosuppressive cells in the TME, including Tregs and tumor-associated macrophages (TAMs), and form a synergistic network to trigger tumor cells evading immune surveillance by limiting the activity of effector T cells (Serrels et al., 2015; Saleh and Elkord, 2019). Aside from that, Tregs within TME can express ICs, which in turn building a positive feedback loop that promotes tumorigenesis and contributes to Treg-mediated acquired resistance against presently authorized ICIs (Saleh and Elkord, 2019). Mast cells density is essential in liver fibrosis and tumor immunology of LIHC (Terada and Matsunaga, 2000), and it works synergistically with Tregs (Ju et al., 2009) in terms of patient survival and prognosis of patients. Moreover, activated neutrophils form extracellular traps that trigger tumorous inflammation and hasten LIHC metastasis (van der Windt et al., 2018). Reversely, Cluster 1/2 are rich in B cell naive, NK cell resting, monocyte, macrophage M1, mast cell activated, showing relatively lower malignant biological behavior. As mentioned above, combining immunotherapeutic approaches with targeted TME might provide insights into optimal personalized medicine of LIHC.

The impressing role of ferroptosis in LIHC has been implicated. Sorafenib, an analogue of ferroptosis inducer erastin, blocks the cystine/glutamate antiporter system (system x_c_^−^), which primarily consists of SLC7A11 (also referred to as xCT) and SLC3A22 subunits, with the exchange of intracellular glutamate and extracellular cysteine, thereby impeding the expression of glutathione and inducing ferroptosis via ROS accumulation (Nie et al., 2018; Xia et al., 2019). According to recent research, SLC7A11 overexpression promotes tumor development by impeding ferroptosis (Dixon et al., 2012; Koppula et al., 2021). Our study also found that the highly expressed SLC7A11 in LIHC patients had adverse prognoses, indicating that these patients were more likely to respond to sorafenib. Additionally, the aforementioned immune cells (Tfh, MCs, TAMs, and neutrophils) associated with poor prognosis were also seen in the high SLC7A11 group. The crosstalk between SLC7A11 and other 32 malignancies with pan-cancer analysis revealed that SLC7A11 showed positive correlations with multiple ICs in most cancer types, but it also predicted a high risk of adverse outcomes in several cancers. Hence, SLC7A11 is expected to be a valuable prognostic signature informing future research into targeting SLC7A11 in cancer immunotherapy except in the LIHC scenario (Cheng et al., 2022).

## Conclusion

LIHC is often diagnosed at an advanced stage with high recurrence rates and limited surgery eligibility, and no optimal treatment is available. In our study, we used the expression differences of FRGs in LIHC to distinguish three different subtypes and their clinical features and prognoses for better risk stratification. Subsequently, we found a robust association between FRGs and immune checkpoint PD-L1 and assessed the heterogeneity of TME among clusters. Among these selected FRGs, SLC7A11 was a unique prognostic biomarker whose high expression level suppressed ferroptosis and predicted unfavorable prognosis but potentially better anti-PD-L1 antibody responsiveness. Similar biological properties of SLC7A11 across cancers have also been detected. Ferroptosis-based immunotherapy has been well opening the door to prosper new avenues for LIHC curative. Nonetheless, further research is required to corroborate the kind of inferences that can be derived from this study.

## Data Availability

The original contributions presented in the study are included in the article/Supplementary Material, further inquiries can be directed to the corresponding author.
