# Supplementary material for: Ferroptosis regulator SLC7A11 is a prognostic marker and correlated with PD-L1 and immune cell infiltration in liver hepatocellular carcinoma
Source: Front Mol Biosci. 2022 Oct 4;9:1012505. doi: 10.3389/fmolb.2022.1012505 (PMC9577028; doi:10.3389/fmolb.2022.1012505)
Supplement: Supplementary file 1 [file DataSheet1.docx]

Supplementary Material

# Supplementary Figures and Table

##
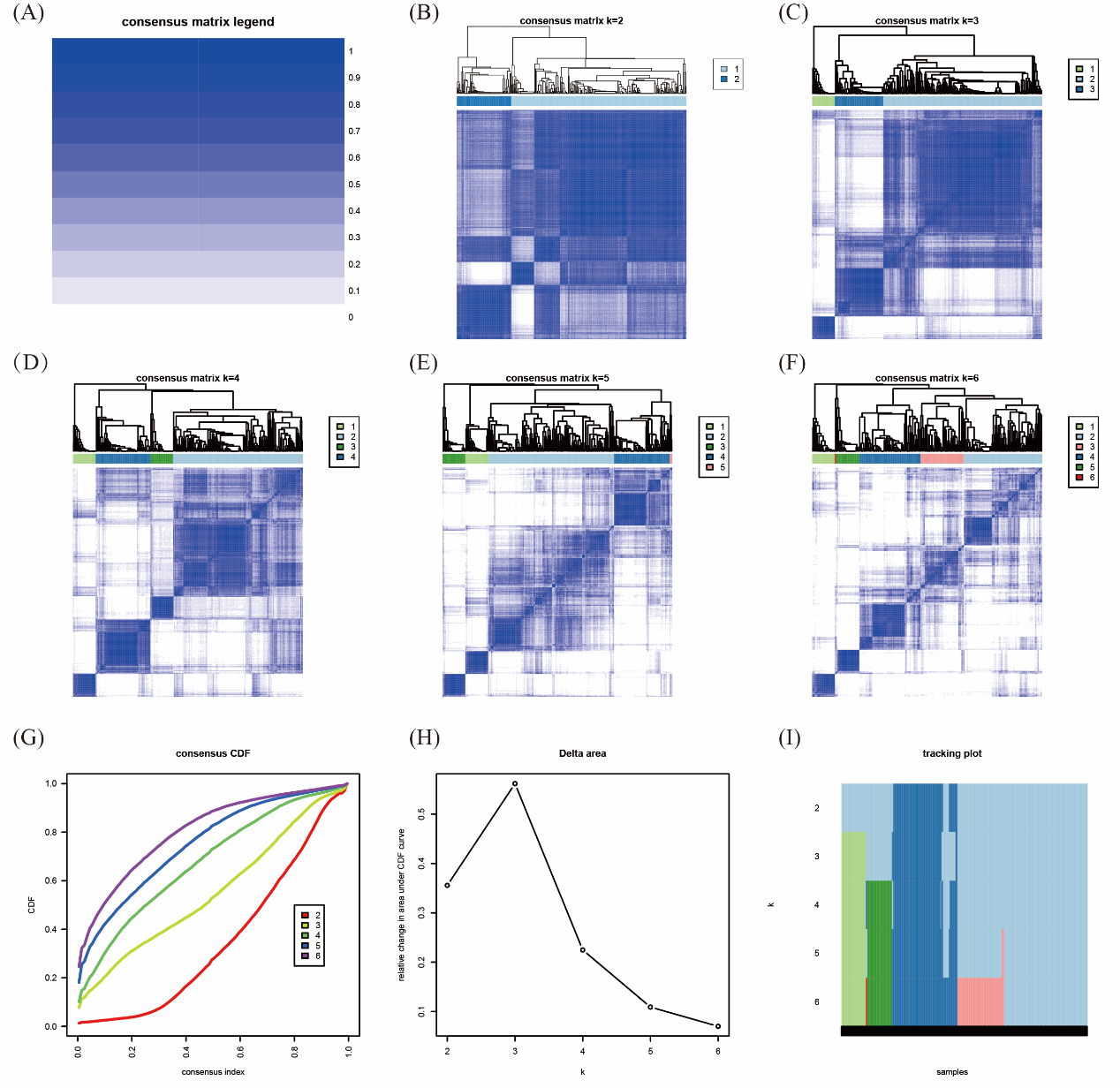
Supplementary Figures

**Supplementary Figure S1 |** Screening process of consistent clustering analysis based on ferroptosis regulators’ expression in tumor and normal samples. **(A)** Consensus matrix legend. **(B-F)** Five heat maps exhibit the clustering matrix for ferroptosis regulators in LIHC patients for k = 2, 3, 4, 5, and 6. **(G)** Cumulative distribution function curves for *k* = 2-6. **(H)** Delta area curve of consensus clustering for *k* = 2-6. **(I)** Tracking plot in the LIHC patients.

**
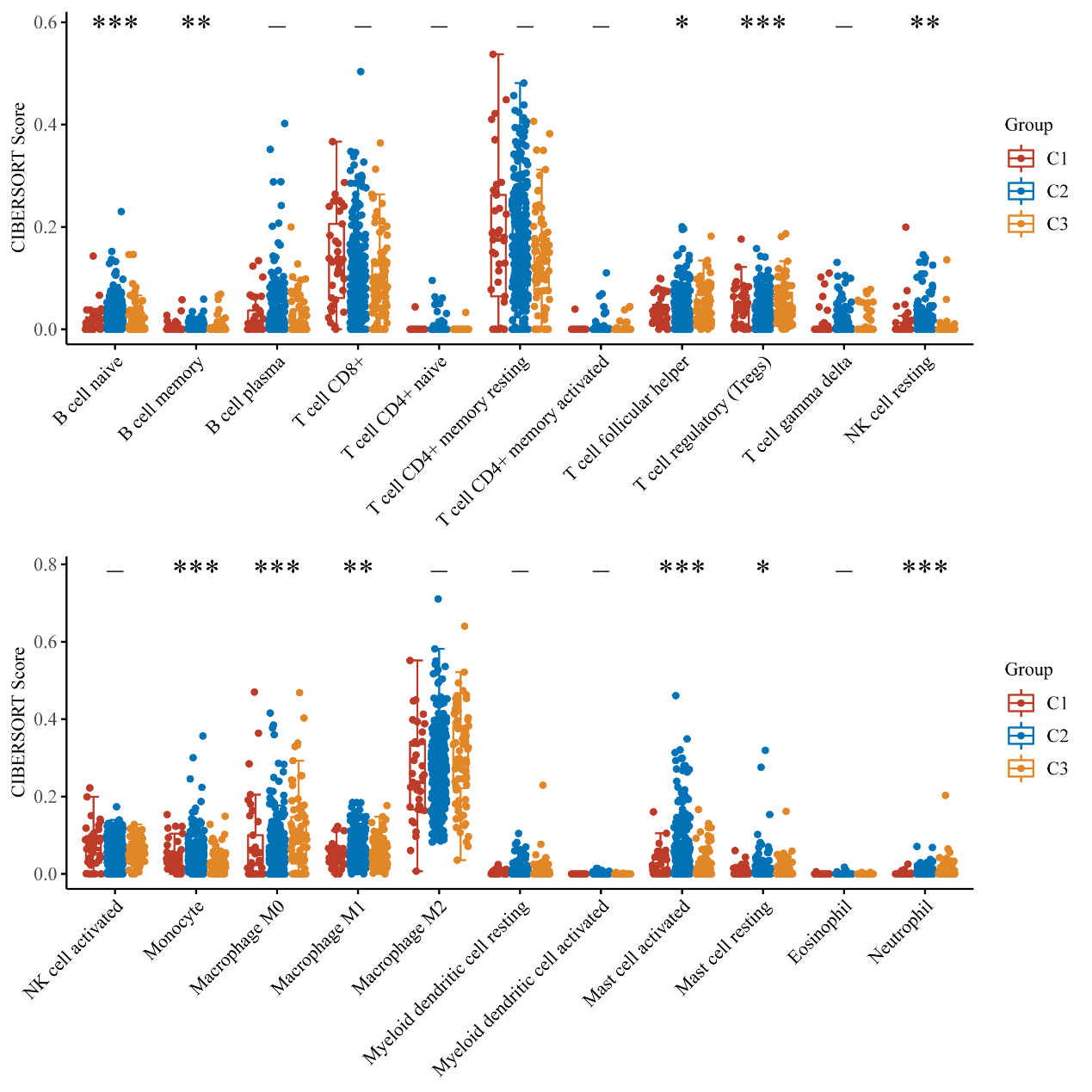
 Supplementary Figure S2 |** Differences in immune cell infiltration of the three subtypes and their scores in CIBERSORT algorithm among 22 immune cells. **p <* 0.05, ***p* < 0.01*, and ***p* < 0.001.

**
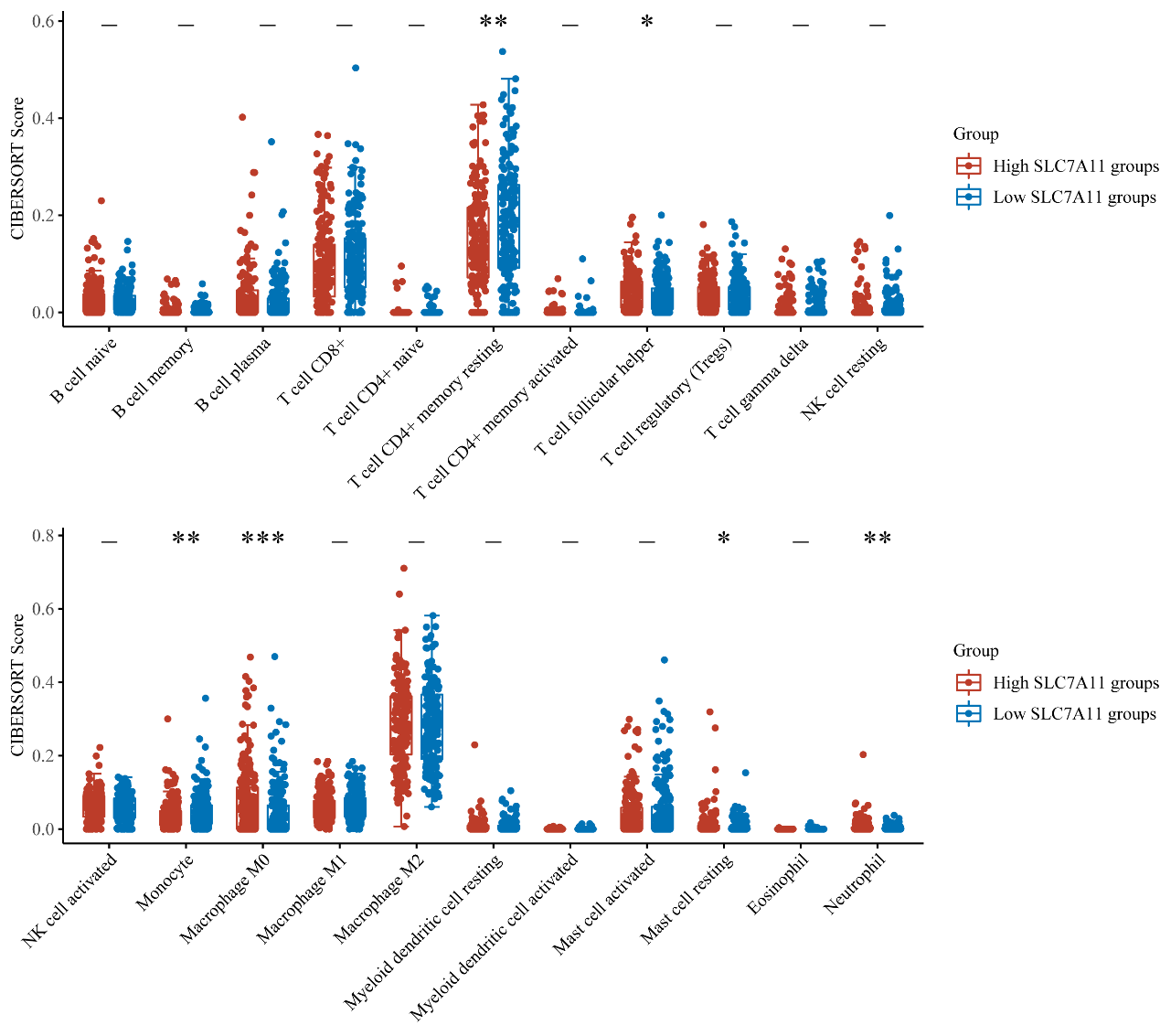
**

**Supplementary Figure S3 |** Differences in immune cell infiltration of the low- and high-SLC7A11 groups and their scores in CIBERSORT algorithm among 22 immune cells. **p <* 0.05, ***p* < 0.01*,* and ****p* < 0.001.

## Supplementary Table

TABLE 1 | Baseline clinical characteristics of LIHC patients in three clusters.

| **Feature** | **Cluster 1** | **Cluster 2** | **Cluster 3** | ***P*_value** |
| --- | --- | --- | --- | --- |
| Alive | 22 | 180 | 39 | 0.003 |
| Dead | 15 | 76 | 39 |  |
| Mean (SD) | 56.5 (13.3) | 60.4 (13.5) | 57.8 (13.4) | 0.046 |
| Median [min, max] | 58 [24, 80] | 62 [16, 90] | 60 [20, 81] |  |
| FEMALE | 11 | 85 | 25 | 0.909 |
| MALE | 26 | 171 | 53 |  |
| AMERICAN INDIAN | 1 |  | 1 |  |
| ASIAN | 19 | 100 | 39 | 0.216 |
| BLACK | 1 | 13 | 3 |  |
| WHITE | 13 | 137 | 34 |  |
| T1 | 18 | 143 | 20 | 0.001 |
| T2 | 13 | 53 | 26 |  |
| T3 | 2 | 27 | 16 |  |
| T3a | 2 | 19 | 8 |  |
| T3b | 1 | 2 | 3 |  |
| T4 | 1 | 8 | 4 |  |
| T2b |  | 1 |  |  |
| TX |  | 1 |  |  |
| T2a |  |  | 1 |  |
| N0 | 25 | 173 | 54 | 0.802 |
| NX | 12 | 81 | 21 |  |
| N1 |  | 2 | 2 |  |
| M0 | 30 | 176 | 60 | 0.137 |
| M1 | 1 | 3 |  |  |
| MX | 6 | 77 | 18 |  |
| I | 18 | 134 | 19 | 0 |
| II | 13 | 50 | 23 |  |
| IIIA | 3 | 39 | 23 |  |
| IIIB | 1 | 4 | 3 |  |
| IIIC | 1 | 4 | 4 |  |
| IV | 1 | 1 |  |  |
| III |  | 3 |  |  |
| IVA |  | 1 |  |  |
| IVB |  | 2 |  |  |
| G1 | 5 | 46 | 4 | 0.001 |
| G2 | 19 | 128 | 30 |  |
| G3 | 10 | 74 | 38 |  |
| G4 | 3 | 4 | 5 |  |
